# Identification of candidate methylation-responsive genes in ovarian cancer

**DOI:** 10.1186/1476-4598-6-10

**Published:** 2007-01-25

**Authors:** Laura Menendez, DeEtte Walker, Lilya V Matyunina, Erin B Dickerson, Nathan J Bowen, Nalini Polavarapu, Benedict B Benigno, John F McDonald

**Affiliations:** 1 Department of Genetics, University of Georgia, Athens, GA 30605, USA; 2 School of Biology, Georgia Institute of Technology, 315 Ferst Drive, Atlanta, GA 30332, USA; 3 Ovarian Cancer Institute, 225 North Ave., Atlanta, GA 30332, USA

## Abstract

**Background:**

Aberrant methylation of gene promoter regions has been linked to changes in gene expression in cancer development and progression. Genes associated with CpG islands (CGIs) are especially prone to methylation, but not all CGI-associated genes display changes in methylation patterns in cancers.

**Results:**

In order to identify genes subject to regulation by methylation, we conducted gene expression profile analyses of an ovarian cancer cell line (OVCAR-3) before and after treatment with the demethylating agent 5-aza-deoxycytidine (5-aza-dC). An overlapping subset of these genes was found to display significant differences in gene expression between normal ovarian surface epithelial cells and malignant cells isolated from ovarian carcinomas. While 40% of all human genes are associated with CGIs, > 94% of the overlapping subset of genes is associated with CGIs. The predicted change in methylation status of genes randomly selected from the overlapping subset was experimentally verified.

**Conclusion:**

We conclude that correlating genes that are upregulated in response to 5-aza-dC treatment of cancer cell lines with genes that are down-regulated in cancer cells may be a useful method to identify genes experiencing epigenetic-mediated changes in expression over cancer development.

## Background

Gene expression profiling is now a common first approach toward the characterization of molecular changes occurring through cancer development and progression [1]. While recurrent changes in patterns of gene expression are beginning to emerge for a variety of cancers, the causal basis of the observed differences in expression remain to be delineated. One characteristic change associated with many cancers is the down regulation of genes involved in suppressing malignant transformation. Down regulation of these "tumor-suppressor genes" may occur, not only by genetic (i.e., nucleotide substitution) changes but also by epigenetic modifications, such as DNA methylation [2,3]. DNA methylation largely occurs at cytosines associated with CpG dinucleotides. CpG rich regions are known as CpG Islands (CGIs). A CGI has been defined as a region of at least 200 bp with a GC content of 50% or more, and an observed/expected ratio of CpGs higher than 0.6 [4]. Methylation of CGIs in the promoter region of genes is known to transcriptionally repress those genes [3]. Numerous reports have shown that multiple genes are silenced during cancer progression through hypermethylation of the CGIs. Some examples of genes shown to be silenced in ovarian cancers due to hypermethylation include *OPCML, RASSF1A, BRAC1 *and *p16 *[5-7].

Treatment of cancer cell lines with the demethylating agent 5-aza-deoxycytidine (5-aza-dC) leads to changes in gene expression due to the loss of methylation in gene regulatory regions [8-11]. In this study, an ovarian cancer cell line (OVCAR-3) was treated with 5-aza-dC to identify genes regulated in cancer cells by methylation. We compared changes in gene expression patterns in the 5-aza-dC treated cancer cell line with observed differences in patterns of gene expression between normal and cancerous ovarian tissue to identify candidate genes undergoing epigenetic changes during the process of ovarian cancer development. The predicted change in methylation status of genes randomly selected from the list of candidate genes was experimentally verified. Our results indicate that correlating genes that are upregulated in response to 5-aza-dC treatment of cancer cell lines with genes that are down-regulated in ovarian cancers may be a useful method to identify genes experiencing epigenetic-mediated changes in expression over ovarian cancer development.

## Results

### Gene expression changes in OVCAR-3 in response to 5-aza-treatment

**Figure 1:**
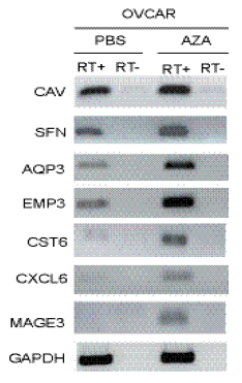
**RT-PCR confirmatory analysis of representative genes shown by microarray analysis to be upregulated after 5-aza-dC treatment of OVCAR-3 cell line**. Expression results from the microarray analysis were confirmed by RT-PCR for *CAV1, SFN, EMP3, CST6, CXCL6 and MAGEA3*. All the genes showed induction of expression after 5-aza-dC treatment (AZA) relative to untreated cells (PBS). + and - indicate the presence or absence of reverse transcriptase during cDNA synthesis. GAPDH was used as an endogenous control.

The ovarian cancer cell line, OVCAR-3, was treated with five μM 5-aza-dC to identify genes that display a change in expression in response to changes in methylation. After 72 hours of treatment, RNA was extracted, used to synthesize biotinylated cRNA, and hybridized to Affymetrix Human U133 Plus 2.0 oligonucleotide arrays representing approximately 47,000 transcripts. GC Robust Multiarray Analysis (GCRMA) signal values were obtained from the .CEL files as normalized data in log2 format. Analysis of variance (ANOVA) was applied to identify differentially expressed genes among the three control and the three 5-aza-dC treated samples. A total of 831 genes were differentially expressed (p < 0.01) between the control and the 5-aza-dC treated cells, of which 465 were upregulated and 366 were down-regulated. Tables 1 and 2 display the 30 genes with the highest fold changes in expression (increase or decrease). A higher fold change in expression was observed for genes upregulated after the treatment possibly due to the direct effect of loss of methylation in gene regulatory regions. Among the genes upregulated after 5-aza-dC treatment were imprinted genes (e.g., *H19*) and genes previously reported to be transcriptionally repressed due to hypermethylation (e.g., *CST6 *and *SPANX *in malignant tissue and *MAGEA 1 *in non-malignant cells/tissues) [12-14]. Confirmation of the microarray results was carried out by RT-PCR of representative genes that were significantly upregulated in response to the 5-aza-dC treatment [*Cystatin 6 *(*CST6*), *Caveolin-1 *(*CAV1*), *Melanoma antigen family A3 *(*MAGEA3*), *Chemokine (C-X-C motif) ligand 6 *(*CXCL6*), *Aquaporin3 *(*AQP3*), *Stratifin *(*SFN*) and *Epithelial membrane protein 3 *(*EMP3*)] (Figure 1).

**Table 1:** Upregulated genes after 5-aza-dC treatment of OVCAR cells. Chromosomal location and fold change in gene expression are shown for 5-aza-dC treated/untreated OVCAR 3 cells.

RefSeq Transcript ID	Symbol	Gene Name	Chr.	Fold Change
NM_001351	DAZL	Deleted in azoospermia-like	3p24	27.52
NM_052957	ACRC	Acidic repeat containing	Xq13	27.07
NM_144594	FLJ32942	Hypothetical protein FLJ32942	12q13	22.73
NM_032858	MAEL	Maelstrom homolog	1q24	18.75
NM_002364	MAGEB2	Melanoma antigen family B, 2	Xp21	18.73
NM_005363	MAGEA6	Melanoma antigen family A, 6	Xq28	16.31
NM_012253	TKTL1	Transketolase-like 1	Xq28	15.02
NM_023930	KCTD14	Potassium channel tetramerisation domain	11q14	11.34
NM_013453	SPANX	Sperm protein associated with the nucleus, X-linked	Xq27	10.99
NM_005362	MAGEA3	Melanoma antigen family A, 3	Xq28	10.56
NM_005335	HCLS1	Hematopoietic cell-specific Lyn substrate 1	3q13	10.41
NM_006228	PNOC	Prepronociceptin	8p21	9.89
NM_152578	FMR1NB	Fragile X mental retardation 1 neighbor	Xq27	9.24
NM_005367	MAGEA12	Melanoma antigen family A, 12	Xq28	8.81
NM_003289	TPM2	Tropomyosin 2 (beta)	9p13	7.00
NM_080618	CTCFL	CCCTC-binding factor (zinc finger protein)-like	20q13	6.39
NM_001011544	MAGEA11	Melanoma antigen family A, 11	Xq28	6.32
NM_005213	CSTA	Cystatin A	3q21	5.80
NR_002196	H19	H19, imprinted maternally expressed untranslated mRNA	11p15	5.71
NM_032048	EMILIN2	Elastin microfibril interfacer 2	18p11	5.41
NM_173571	LOC255313	Hypothetical protein LOC255313	Xq24	5.28
NM_003480	MFAP5	Microfibrillar associated protein 5	12p13	4.79
NM_001323	CST6	Cystatin E/M	11q13	4.79
NM_001327	CTAG1B&A	Cancer-testis antigen 1B/cancer-testis antigen 1A	Xq28	4.58
NM_001011548	MAGEA4	Melanoma antigen family A, 4	Xq28	4.48
NM_020826	SYT13	Synaptotagmin XIII	11p11	4.37
NM_013238	DNAJC15	DnaJ (Hsp40) homolog, subfamily C, member 15	13q14	4.33
NM_005602	CLDN11	Claudin 11	3q26	4.23
NM_001002915	IGFL2	Insulin growth factor-like family member 2	19q13	4.09
NM_004988	MAGEA1	Melanoma antigen family A, 1 (directs expression of antigen MZ2-E)	Xq28	3.73

**Table 2:** Downregulated genes after 5-aza-dC treatment of OVCAR cells. Chromosomal location and fold change in gene expression are shown for 5-aza-dC treated/untreated OVCAR 3 cells.

RefSeq Transcript ID	Symbol	Gene Name	Chr.	Fold Change
NM_0010138360	MAD1L1	MAD1 mitotic arrest deficient-like 1 (yeast)	7p22	-4.27
NM_004883	NRG2	Neuregulin 2	5q31	-3.95
NM_001001331	ATP2B2	ATPase, Ca++ transporting, plasma membrane 2	3p25	-2.88
NM_001719	BMP7	Bone morphogenetic protein 7 (osteogenic protein 1)	20q13	-2.56
NM_032932	RAB11FIP4	RAB11 family interacting protein 4 (class II)	17q11	-2.49
NM_014914	CENTG2	Centaurin, gamma 2	2q37	-2.30
NM_013372	GREM1	Gremlin 1, cysteine knot superfamily, homolog	15q13	-2.28
XM_373734	---	Hypothetical LOC388388	3p13	-2.28
NM_020761	Raptor	Raptor	17q25.3	-2.26
NM_001031693	HHLA3	HERV-H LTR-associating 3	---	-2.20
NM_003598	TEAD2	TEA domain family member 2	19q13	-2.18
NM_022773	FLJ12681	Hypothetical protein FLJ12681	16p13.3	-2.18
NM_004389	CTNNA2	Catenin (cadherin-associated protein), alpha 2	2p12	-2.09
XM_032996	MICAL3	Microtubule associated monoxygenase, calponin and LIM domain containing 3	22q11	-2.08
NM_000620	NOS1	Nitric oxide synthase 1 (neuronal)	12q24	-2.06
NM_006567	FARS2	Phenylalanine-tRNA synthetase 2	6p25	-1.20
NM_003466	PAX8	Paired box 8	2q13	-1.99
NM_006613	GRAP	GRB2-related adaptor protein-like	17p11	-1.97
NM_022138	SMOC2	SPARC related modular calcium binding 2	6q27	-1.96
NM_017594	DIRAS2	DIRAS family, GTP-binding RAS-like 2	9q22	-1.96
NM_021018	HIST1H3F	Histone 1, H3f	6p22	-1.92
NM_004192	ASMTL	Acetylserotonin O-methyltransferase-like	Xp22	-1.92
NM_002412	MGMT	O-6-methylguanine-DNA methyltransferase	10q26	-1.91
NM_001466	FZD2	Frizzled homolog 2 (Drosophila)	17q21	-1.91
XM_498471	LOC147727	Hypothetical protein LOC147727	19p13	-1.90
NM_003562	SLC25A11	Solute carrier family 25 (mitochondrial carrier; oxoglutarate carrier), member 11	17p13	-1.88
NM_004565	PEX14	Peroxisomal biogenesis factor 14	1p36	-1.87
NM_003955	SOCS3	Suppressor of cytokine signaling 3	17q25	-1.87
NM_007037	ADAMTS8	ADAM metallopeptidase with thrombospondin type 1 motif, 8	11q24	-1.85
NM_022479	WBSCR17	Williams-Beuren syndrome chromosome region 17	7q11	-1.84

### Methylation pattern of candidate genes

Genes displaying altered expression levels after 5-aza-dC treatment could have been directly or indirectly affected by loss of methylation. Those genes directly affected by 5-aza-dC would be expected to have a methylated CGI in or in proximity to promoter regions. Indirect effects might include the altered expression of loci regulated by genes containing CGIs. To identify genes associated with CGIs, we utilized the CGI identification feature of the USCS Human Genome Browser Gateway. Of the 831 genes differentially expressed after 5-aza-dC treatment, 74% are known to be associated with CGIs.

The methylation pattern of the promoter region of five genes associated with CGIs and found to be upregulated after 5-aza-dC treatment (*CAV1, CST6, MAGEA3, EMP3 *and *CXCL6*) was examined. With the exception of *CXCL6*, all have been previously shown to be hypermethylated in other cancers or cancer cell lines [12,15-17].

**Figure 2:**
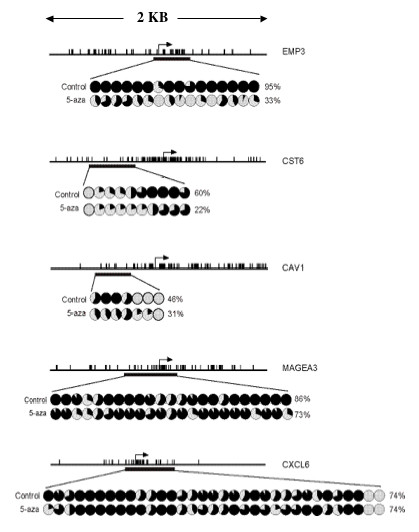
**Methylation analysis of genes upregulated after 5-aza-dC treatment of the OVCAR-3 cell line**. Summary of sodium bisulfite sequencing analysis of CGIs associated with the *EMP3, CST6, CAV1, MAGEA3 *and *CXCL6 *genes in 5-aza-dC treated and untreated (control) OVCAR-3 cells. Five clones were sequenced for each sample, and each circle displays the percent methylation of all clones for a single CpG dinucleotide (open circle, 100% unmethylated; filled circle, 100% methylated). Average percent methylation across all CpG sites for untreated (control) and 5-aza-dC treated cells is shown next to each row of circles. (*Vertical bars*, CpG dinucleotides; *arrow*, position of the transcription start site; *horizontal black rectangle*, region amplified and sequenced after sodium bisulfite).

Figure 2 presents the results of sodium bisulfite sequencing analysis of CpG rich regulatory regions in each of the five genes. All genes showed extensive methylation of their promoter regions in the untreated ovarian cancer cells (OVCAR-3) and a variable decrease of methylation after 5-aza-dC treatment. *CST6*,*CAV1*,*EMP3 *and *MAGEA3 *displayed a decrease in methylation ranging from 13% to 61% while *CXCL6 *exhibited no methylation change in the promoter region examined. Variability in the response of different CpG regions to 5-aza-dC has been observed previously [9,18], therefore we can not exclude the possibility that expression of *CXCL6 *is controlled by the methylation levels of a different region than the one studied here.

### Genes responsive to 5-aza-dC in the OVCAR-3 cell line display decreased expression in ovarian adenocarcinomas

We hypothesized that if epigenetic mediated changes in gene expression are occurring in ovarian cancer development, at least a subset of the genes upregulated in response to 5-aza-dC treatment in OVCAR-3 cells should display lower levels of expression in ovarian adenocarcinomas relative to controls. To test this hypothesis, we looked for overlap between genes responsive to 5-aza-dC treatment in the OVCAR-3 cells and genes displaying a significant change in expression between normal ovarian surface epithelium brushings (NOSE) and epithelial adenocarcinoma cells isolated from serous papillary epithelial ovarian tumors (EOC) by laser capture microdissection (LCM). Microarray analysis (Affymetrix U133 Plus 2.0) was performed on eight samples of NOSE brushings and eight LCM collections of epithelial cells from EOC. Differentially expressed genes between these samples were identified as described for the OVCAR-3 cell line experiment. Although a comprehensive analysis of the tissue microarray results will be published elsewhere, the average fold difference in expression levels between NOSE and EOC for the overlapping genes (see below) are presented in Additional File 1.

Of the 831 genes displaying a significant change in expression in response to 5-aza-dC treatment of OVCAR-3 cells, 123 displayed a significant decrease in expression between normal ovarian epithelia and adenocarcinomas [see Additional File 1]. Of these 123 genes, 102 were upregulated and 21 were down-regulated in response to the 5-aza-dC treatment in the OVCAR-3 cell line. Included in the list of 102 genes are genes previously identified as being hypermethylated and down-regulated in cancer (e.g., *zinc finger protein 185 *in prostate cancer [19], *CAV1 *in breast cancer [20], and *DIRAS3 *in ovarian cancer [21]). We thus predicted that the 102 genes upregulated in 5-aza-dC treated cells that overlapped with genes down-regulated in EOC were likely to have been so, at least in part, due to hypermethylation. To determine if these 102 candidate genes contained CGIs as our model predicts, we examined sequences in and immediately flanking each of the 102 genes. Ninety-six of the 102 candidate genes (94 %) were found to have CGIs. In contrast, it has been estimated that only 40% of all human genes are associated with CGIs [22].

**Figure 3:**
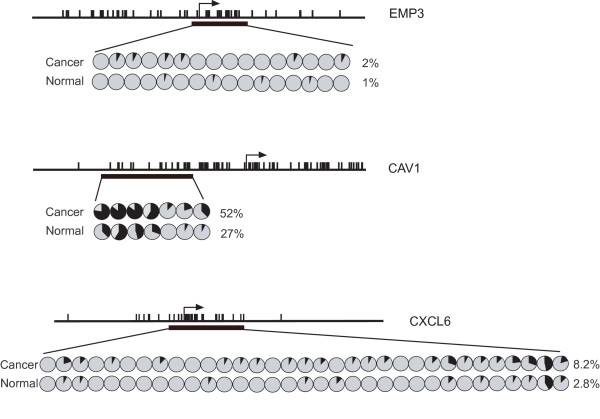
**Methylation analysis of the *EMP3, CAV1 *and *CXCL6 *genes in ovarian cancer (EOC) and control (NOSE) tissues**. Summary of sodium bisulfite sequencing analysis of CGIs associated with the *EMP3, CAV1 *and *CXCL6 *genes in ovarian ovarian cancer and control (NOSE) tissues. For *EMP3*, 16 clones for 4 cancer samples and 23 clones for 5 control (NOSE) samples were sequenced. For *CAV 1*, 17 clones for 4 cancer samples and 18 clones for 4 control (NOSE) samples were sequenced. For *CXCL6*, 19 clones for 4 cancer samples and 24 clones for 5 normal (NOSE) samples were sequenced. Each circle displays the percent methylation of all clones for a single CpG dinucleotide (open circle, 100% unmethylated; filled circle, 100% methylated). Average percent methylation for cancer (EOS) and control (NOSE) samples is shown next to each row of circles. (*Vertical bars*, CpG dinucleotides; *arrow*, position of the transcription start site; *horizontal black rectangle*, region amplified and sequenced after sodium bisulfite).

As an initial experimental test of the model, we randomly selected two genes from the list of candidate genes (*CAV 1 *and *CXCL6*) and one CGI-associated gene not on the list of candidate genes (*EMP3*) for sodium bisulfite sequencing analysis. Consistent with the prediction of the model, *CAV-1 *and *CXCL6 *were found to be hypermethylated in ovarian cancer relative to control tissues while essentially no difference in *EMP3 *methylation pattern was detected. The observed fold changes in gene expression between control (NOSE) and ovarian cancer (EOC) samples were significant (p < 0.01) for *CAV 1 *(-30.88) and *CXCL6 *(-4.22) [see Additional File 1] but not significant (p < 0.06) for *EMP 3 *(+1.10). Consistent with these relative changes in levels of gene expression, the relative increase in levels of methylation was greater for *Cav 1 *than *CXCL6 *and minimal for *EMP3 *(Figure 3).

## Discussion

The potential contribution of epigenetic changes to cancer development and progression is a topic of much recent interest [e.g., [23-25]]. For example, several studies have implicated changes in DNA methylation with changes in the expression of a number of putative tumor suppressor and oncogenes in a variety of cancers [e.g., [2,12-21]]. In an effort to establish a more global perspective of genes that may be prone to changes in DNA methylation during cancer development, a number of investigators have carried out gene profile analyses of a variety of cancer cells treated with demethylating agents (e.g., 5-aza-dC) [e.g., [8-11,26-28]]. The rationale is that genes that are transcriptionally repressed in cancer cells due to hypermethlyation may be released from such constraint after treatment with demethylating agents and thereby identified by a significant increase in gene expression. If valid, such a strategy could be useful in identifying a candidate subset of methylation-regulated genes among the thousands of genes that are typically found to be down-regulated during cancer development. While intuitively appealing, such a strategy rests upon the questionable assumption that all or most genes displaying a significant increase in expression after treatment with demethylating agents are directly responding to an induced change in methylation. The purpose of our study was to conduct a preliminary evaluation of the validity of this assumption and of the overall utility of this approach within the context of ovarian cancer.

We selected for our study the ovarian cancer cell line OVCAR 3 because we had previously determined that it displays a gene expression profile most similar to that of ovarian cancer epithelial cells (data not shown). Comparing the gene expression profile of OVCAR 3 cells before and after treatment with 5-aza-dC revealed a significant (p < 0.01) change in expression for 831 genes. We determined that 615 of these 831 genes (74%) are associated with CpG islands (CGIs). This is a substantially higher fraction than what has been estimated for the human genome overall (40%) [22], and suggests that, as expected, CGI-associated genes are especially sensitive to 5-aza-dC treatment. Only slightly more than half (465 or 56%) of the 831 5-aza-dC-responsive genes were significantly upregulated after 5-aza-dC treatment as would be expected if they had been hypermethylated in the cancer cell. The explanation as to why the remaining 366 genes displayed a significant decrease in expression after 5-aza-dC treatment is unknown but could, in part, be due to hypomethylated CpG-associated genes being accessed by repressor proteins or to indirect regulatory effects (e.g., non-CpG-associated genes responding to an upregulated repressor protein) or to some unknown gene toxic effect of the treatment. Regardless, we can conclude that up to nearly half of the 5-aza-dC responsive genes in the OVCAR 3 cells may not have been directly responding to 5-aza-dC-induced hypomethylation. On the other hand, support for the hypothesis that the majority of those genes upregulated in the cell line after treatment are directly responding to 5-aza-dC-induced hypomethylation comes from the fact that 5 of the 6 upregulated genes that we directly tested were indeed hypomethylated in response to the 5-aza-dC treatment.

To evaluate the possible relevance of the results from the cell line experiment to actual changes in gene expression that occur during ovarian cancer development, we correlated the OVCAR 3 expression profiles with those from an ovarian cancer tissue analysis recently completed in our laboratory. We reasoned that if methylation dependent changes in gene expression are occurring in ovarian cancer development, at least a subset of the genes upregulated in response to 5-aza-dC treatment in OVCAR 3 cells should display lower levels of expression in ovarian cancer cells relative to controls. Of the 465 genes upregulated in the OVCAR 3 experiment, 102 (22%) were found to be down-regulated in the ovarian cancer cells isolated from tumor samples. Since 102 genes is a small fraction (~0.02 %) of the ~5000 genes that were down-regulated in ovarian cancer cells and that we estimate that nearly half of these are associated with CGIs (data not shown), it seems unlikely that monitoring the gene expression response of OVCAR 3 cells to 5-aza-dC is an effective way to identify all candidate genes that may experience functionally significant changes in methylation over cancer development. However, we believe the approach may be useful in identifying a subset of genes that are most likely to experience functionally significant changes in methylation over cancer development. This approach can provide researchers with a useful method by which to identify a candidate set of genes for detailed epigenetic analysis. Our methylation analysis of randomly selected genes from the list of 102 overlapping genes, supports our conclusion.

## Conclusion

We conclude that correlating genes that are upregulated in response to 5-aza-dC treatment of cancer cell lines with genes that are down-regulated in cancer cells may be a useful method to identify genes experiencing epigenetic-mediated changes in expression over cancer development.

## Methods

### Cell lines and cell culture

OVCAR-3 cells were grown in RPMI 1640 supplemented with 20% v/v heat-inactivated FBS (Invitrogen, Carlsbad, CA), 2 mM L-glutamine (Mediatech Inc., Herndon, VA), 10 mM HEPES buffer (Sigma, St. Louis, MO) penicillin (100 U/ml), streptomycin (100 μg/ml), 1 mM sodium pyruvate (Mediatech Inc.), and 0.01 mg/ml bovine insulin (Sigma).

### 5-aza-dC treatment

OVCAR-3 cells were plated at a concentration of 1 × 10^6 ^in 10 cm^3 ^cell culture dishes. Cells were allowed to adhere to the plates overnight, and the medium was replaced the next morning. Five μM 5-aza-deoxycytidine (Sigma) or PBS was added to the medium and the plates were incubated for 3 days at 37°C in a 5% CO^2 ^atmosphere. Each treatment was done in triplicate.

### Microarray

OVCAR-3 cells were washed once in the plate with HBSS prior to the extraction of RNA and DNA by established techniques (Trizol). The integrity of the RNA was verified using an Agilent 2100 Bioanalyzer (Agilent Technologies, Palo Alto, CA). Biotin labeled cRNA was synthesized, hybridized to Affymetrix U133 Plus 2.0 oligonucleotide arrays and analyzed with a GeneChip Scanner 3000 (Affymetrix, Santa Clara, CA).

### Microarray data analysis

CEL files generated by the Affymetrix Gene Chip Operating System (GCOS) were converted to expression level values using the GCRMA version1.1.0 (ref. below) package implemented from within the Spotfire DecisionSite for Microarray Analysis (DSMA). Probe set intensities were filtered with DSMA using a modulation threshold of 5 to include only those probe sets with at least a log base 2 expression value of ≥ 5. Differentially expressed probe sets were identified using the t-test function of the Profile Anova Tool of DSMA. SAM (Significance Analysis of Microarrays v.2.21) was used to calculate fold change values for the differentially expressed probe sets [29]. Annotations for probe sets were obtained from the NetAffx website [30].

### RT-PCR analysis

1 μg of total RNA was reverse transcribed using oligo (dT) primers from the Superscript III cDNA synthesis kit (Invitrogen). PCR was performed in an Opticon 2 (MJ Research) using 1 μl of synthesized cDNA. Primer sequences and expected length of the products are provided in Table 3. Gene expression was normalized to the expression of the housekeeping gene GAPDH.

**Table 3:** Primers used for RT-PCR and Sodium Bisulfite sequencing

Gene	Primer	Sequence	Size	Tm	Ref.
CAV1	RT-PCR_F	5'-CGACCCTAAACACCTCAACGATG-3'	278	55°C	[15]
	RT-PCR-R	5'-GCAGACAGCAAGCGGTAAAACC-3'			
	MOD_F	5'-TGTGTATTTTGTAAATATGGTATAATTTG-3'	537	58°C	[15]
	MOD_R	5'-CATTTTCCCTACTCTAAACCAC-3'			
					
SFN	RT-PCR_F	5'-GTGTGTCCCCAGAGCCATGG-3'	161	60°C	[23]
	RT-PCR-R	5'-ACCTTCTCCCGGTACTCACG-3'			
					
CST6	RT-PCR_F	5'-AAGACCAGGGTCACTGGAGA-3'	163	60°C	[12]
	RT-PCR-R	5'-CGGGGACTTATCACATCTGC-3'			
	MOD_F	5'-GGTTGGAATGTTGTAGTGGT-3'	430	55°C	[12]
	MOD_R	5'-CCCCAACAACAAATACCAA-3'			
					
MAGEA3	RT-PCR_F	5'-TGGAGGACCAGAGGCCCCC-3'	725	68°C	[16]
	RT-PCR-R	5'-GGACGATTATCAGGAGGCCTGC-3'			
	MOD_F	5'-AGATTTGGTTTGAGGGGAGTAGAAGT-3'	405	60°C	[16]
	MOD_R	5'-AACCC(G/A)ACAACAAAAACAACACTAAA-3'			
					
EMP3	RT-PCR-F	5'-TGCTCTCCCTCATTCTCTGCTGTC-3'	274	60°C	
	RT-PCR-R	5'-CGCTTCCGTAGGTGGATGTAGATG-3'			
	MOD-F	5'-TAGTATATATTGAGAGGAGGAGAG-3'	340	62°C	[17]
	MOD-R	5'-CTTCCCAAACTACTACATTCCCA-3'			
					
CXCL6	RT-PCR-F	5'-CCTGAAGAACGGGAAGC- 3'	135	60°C	
	RT-PCR-R	5'-GACTGGGCAATTTTATGATG-3'			
	MOD-F	5'-GAGGGATGAATGTAGATAAAGGGAGT-3'	616	60°C	
	MOD-R	5'-AACTTCCAAATCCAAACAAACTTACTT-3'			
					
GAPDH	RT-PCR-F	5'-GAAATCCCATCACCATCTTCCAG-3'	312	56°C	
	RT-PCR-R	5'-ATGAGTCCTTCCACGATACAAAAG-3'			

Size, product size in bp; Tm, annealing temperature

### DNA methylation analysis

Genomic DNA, from 5-aza-dC treated and control OVCAR 3 cells and NOSE brushings and ovarian cancer tissues, was treated with sodium bisulfite to convert unmethylated cytosines to thymines. The EZ DNA Methylation-Gold Kit (Zymo, Orange, CA) was used to modify 500 ng to 1 μg of DNA. PCR conditions and primer sequences are provided in Table 3. PCR fragments were gel extracted using the Tissue DNA Extraction Kit (Qiagen) and cloned into pCR 2.1-TOPO TA cloning vectors (Invitrogen). For the OVCAR 3 treated and untreated cell lines, 5 clones each were extracted using the Qiaprep Spin Miniprep Kit (Qiagen) and sequenced (University of Georgia, DNA sequencing Facility). Eighteen to 24 clones from 4 × 5 NOSE (control) samples and 16–19 clones from four cancer samples were sequenced by High-Throughput Sequencing Solutions at the University of Washington.

## Competing interests

The author(s) declare that they have no competing interests.

## Authors' contributions

LM carried out the methylation analyses and RNA extractions of OVCAR-3 cells, participated in the 5-aza-dC treatments and the microarray data analyses and drafted the manuscript. DW carried out the methylation analyses on normal (NOSE) and ovarian cancer (EOC) samples and assisted in the draft of the manuscript. LVM conducted the microarray analyses, EBD participated in the cell culture and 5-aza-dC treatments. NJB contributed to the design of the study and participated in the microarray data analyses. NP contributed to the bioinformatics analysis of the data. BBB provided the ovarian tissue samples. JFM contributed to the design and coordination of the study and composed the final draft of the manuscript. All authors read and approved the final manuscript.

## Supplementary Material

Additional file 1 Overlap between genes displaying a significant (p < 0.01) changes of expression in OVCAR-3 after 5-aza-dC treatment and genes down-regulated in ovarian cancer tissue samples. Fold change in gene expression is shown for 5-aza-dC treated relative to untreated OVCAR 3 cells and for ovarian cancer (EOC) relative to control (NOSE) patient samples. Also shown is the chromosomal location of each gene (Chr). Click here for file
