# Supplementary material for: Identification of candidate methylation-responsive genes in ovarian cancer
Source: Mol Cancer. 2007 Jan 25;6:10. doi: 10.1186/1476-4598-6-10 (PMC1803786; doi:10.1186/1476-4598-6-10)
Supplement: Additional file 1 — Overlap between genes displaying a significant (p < 0.01) changes of expression in OVCAR-3 after 5-aza-dC treatment and genes down-regulated in ovarian cancer tissue samples. Fold change in gene expression is shown for 5-aza-dC treated relative to untreated OVCAR 3 cells and for ovarian cancer (EOC) relative to control (NOSE) patient samples. Also shown is the chromosomal location of each gene (Chr). [file 1476-4598-6-10-S1.doc]

| RefSeq  Transcript ID | | | Symbol | | Gene Name | Cell line  Fold change | Tissue  Fold change | Chr |
| --- | --- | --- | --- | --- | --- | --- | --- | --- |
| NM_013453 | | | SPANX | | Sperm protein associated with the nucleus, X-linked, family member A1 | 10.99 | -8.58 | Xq27 |
| NM_001323 | | | CST6 | | Cystatin E/M | 4.79 | -2.16 | 11q13 |
| NM_002993 | | | CXCL6 | | Chemokine (C-X-C motif) ligand 6 | 3.10 | -4.22 | 4q13 |
| NM_024829 | | | FLJ22662 | | Hypothetical protein FLJ22662 | 2.97 | -3.97 | 12p13 |
| NM_001482 | | | GATM | | glycine amidinotransferase | 2.96 | -7.49 | 15q21 |
| NM_005261 | | | GEM | | GTP binding protein overexpressed in skeletal muscle | 2.54 | -1.90 | 8q22 |
| NM_004925 | | | AQP3 | | Aquaporin 3 | 2.51 | -3.31 | 9p13 |
| NM_003982 | | | SLC7A7 | | Solute carrier family 7 (cationic amino acid transporter, y+ system), member 7 | 2.47 | -2.52 | 14q11 |
| NM_001033910 | | | TRAF5 | | TNF receptor-associated factor | 2.36 | -2.38 | 1q32 |
| NM_002526 | | | NT5E | | 5'-nucleotidase, ecto (CD73) | 2.25 | -16.40 | 6q14 |
| NM_001003818 | | | TRIM6 | | Tripartite motif-containing 6 | 2.14 | -7.35 | 11p15 |
| NM_024642 | | | GALNT12 | | UDP-N-acetyl-alpha-D-galactosamine: | 2.00 | -3.60 | 9q22 |
| NM_001122 | | | ADFP | | Adipose differentiation-related protein | 1.99 | -8.49 | 9p22 |
| NM_021199 | | | SQRDL | | Sulfide quinone reductase-like (yeast) | 1.96 | -5.91 | 15q21 |
| XM_374020 | | | LOC375295 | | Hypothetical gene supported by BC013438 | 1.95 | -10.75 | 2q31 |
| NM_001006605 | | | FAM69A | | Family with sequence similarity 69, member A | 1.92 | -4.37 | 1p22 |
| NM_002210 | | | ITGAV | | Integrin, alpha V | 1.92 | -6.22 | 2q32 |
| NM_001753 | | | CAV1 | | Caveolin 1, caveolae protein, 22kDa | 1.87 | -30.88 | 7q31 |
| NM_006404 | | | PROCR | | Protein C receptor, endothelial (EPCR) | 1.80 | -12.18 | 20q11 |
| NM_004675 | | | DIRAS3 | | DIRAS family, GTP-binding RAS-like 3 | 1.76 | -6.31 | 1p31 |
| NM_001860 | | | SLC31A2 | | Solute carrier family 31 (copper transporters), member 2 | 1.73 | -8.57 | 9q32 |
| NM_021979 | | | HSPA2 | | Heat shock 70kDa protein 2 | 1.73 | -10.90 | 14q23 |
| NM_016441 | | | CRIM1 | | Cysteine rich transmembrane BMP regulator 1 (chordin-like) | 1.72 | -6.92 | 2p22 |
| NM_152611 | | | C20orf75 | | Chromosome 20 open reading frame 75 | 1.72 | -15.61 | 20p12 |
| NM_006283 | | | TACC1 | | Transforming acidic coiled-coil containing protein 1 | 1.65 | -12.00 | 8p11 |
| NM_001004051 | | | GPRASP2 | | G protein-coupled receptor associated sorting protein 2 | 1.63 | -10.21 | Xq22 |
| XM_172341 | | | ZBTB38 | | Zinc finger and BTB domain containing 38 | 1.62 | -2.70 | 3q23 |
| NM_018424 | | EPB41L4B | | | Erythrocyte membrane protein band 4.1 like 4B | 1.62 | -3.97 | 9q31 |
| NM_000345 | | | SNCA | | Synuclein, alpha | 1.61 | -11.43 | 4q22 |
| NM_003115 | | | UAP1 | | UDP-N-acteylglucosamine pyrophosphorylase 1 | 1.61 | -4.90 | 1q23.3 |
| NM_006868 | | | RAB31 | | RAB31, member RAS oncogene family | 1.60 | -5.50 | 18p11 |
| NM_080821 | | C20orf108 | | | Chromosome 20 open reading frame 108 | 1.60 | -3.49 | 20q13 |
| NM_000147 | | | FUCA1 | | Fucosidase, alpha-L- 1, tissue | 1.58 | -3.50 | 1p36 |
| NM_020828 | | | ZFP28 | | Zinc finger protein 28 homolog (mouse) | 1.57 | -3.68 | 19q13 |
| XM_091914 | | LOC162993 | | | Hypothetical protein LOC162993 | 1.55 | -1.80 | 19 p13 |
| NM_024616 | | | TTMP | | TPA-induced transmembrane protein | 1.54 | -2.58 | 3q13 |
| NM_004789 | | | LHX2 | | LIM homeobox 2 | 1.53 | -68.75 | 9q33 |
| NM_014570 | | ARFGAP3 | | | ADP-ribosylation factor GTPase activating protein 3 | 1.53 | -4.27 | 22q13 |
| NM_031412 | GABARAPL | | | | GABA(A) receptor-associated protein like | 1.53 | -5.23 | 12q26 |
| NM_007216 | | | HPS5 | | Hermansky-Pudlak syndrome 5 | 1.53 | -2.72 | 11p15 |
| NM_007223 | | | GPR | | Putative G protein coupled receptor | 1.52 | -3.15 | 15q14 |
| NM_017526 | | | LEPROT | | Leptin receptor overlapping transcript | 1.49 | -8.29 | 1p31 |
| NM_133636 | | | HEL308 | | DNA helicase HEL308 | 1.48 | -3.50 | 4q21 |
| NM_022157 | | | RRAGC | | Ras-related GTP binding C | 1.48 | -2.75 | 1p34 |
| NM_001012978 | | | NGFRAP1L1 | | NGFRAP1-like 1 | 1.48 | -6.18 | Xq22 |
| XM_291277 | | | DKFZp761P0423 | | Hypothetical protein DKFZp761P0423 | 1.47 | -2.84 | 8p23 |
| NM_004235 | | KLF4 | | | Kruppel-like factor 4 (gut) | 1.47 | -8.60 | 9q31 |
| NM_014504 | | RABGEF1 | | | RAB guanine nucleotide exchange factor (GEF) 1 | 1.45 | -1.82 | 7q11 |
| NM_007044 | | | KATNA1 | | Katanin p60 (ATPase-containing) subunit A 1 | 1.45 | -5.84 | 6q25 |
| NM_014817 | | KIAA0644 | | | KIAA0644 gene product | 1.45 | -2.50 | 7p15 |
| NM_022482 | | | ZNF336 | | Zinc finger protein 336 | 1.44 | -2.60 | 20p11 |
| NM_007150 | | | ZNF185 | | Zinc finger protein 185 (LIM domain) | 1.43 | -3.24 | Xq28 |
| NM_015339 | | | ADNP | | Activity-dependent neuroprotector | 1.43 | -3.26 | 20q13 |
| NM_006493 | | | CLN5 | | Ceroid-lipofuscinosis, neuronal 5 | 1.43 | -5.42 | 13q22 |
| NM_013279 | | | C11orf9 | | Chromosome 11 open reading frame 9 | 1.41 | -2.22 | 11q12 |
| NM_005749 | | | TOB1 | | Transducer of ERBB2, 1 | 1.41 | -3.89 | 17q21 |
| NM_007085 | | | FSTL1 | | Follistatin-like 1 | 1.40 | -6.47 | 3q13 |
| NM_004105 | | | EFEMP1 | | EGF-containing fibulin-like extracellular matrix protein 1 | 1.39 | -83.52 | 2p16 |
| NM_003690 | | | PRKRA | | Protein kinase, interferon-inducible double stranded RNA dependent activator | 1.38 | -1.75 | 2q31 |
| NM_015251 | | | ASCIZ | | ATM/ATR-Substrate Chk2-Interacting Zn2+-finger protein | 1.38 | -2.79 | 16q23 |
| NM_016545 | | | IER5 | | Iimmediate early response 5 | 1.37 | -4.47 | 1q25 |
| NM_012406 | | | PRDM4 | | PR domain containing 4 | 1.37 | -2.03 | 12q23 |
| NM_030759 | | | NRBF2 | | Nuclear receptor binding factor 2 | 1.36 | -3.27 | 10q21 |
| NM_019592 | | | RNF20 | | Ring finger protein 20 | 1.36 | -2.80 | 9q31 |
| NM_152435 | | MGC35366 | | | Hypothetical protein MGC35366 | 1.35 | -3.63 | 12q23 |
| NM_002306 | | | LGALS3 | | Lectin, galactoside-binding, soluble, 3 (galectin 3) | 1.35 | -8.92 | 14q22 |
| NM_015391 | | | ANAPC13 | | Anaphase promoting complex subunit 13 | 1.34 | -3.80 | 3q22 |
| NM_020781 | | | ZNF398 | | Zinc finger protein 398 | 1.32 | -3.23 | 7q36 |
| NM_018428 | | | C17orf40 | | Chromosome 17 open reading frame 40 | 1.32 | -2.22 | 17q11 |
| NM_016548 | | | GOLPH2 | | golgi phosphoprotein 2 | 1.31 | -8.59 | 9q21 |
| NM_003340 | | | UBE2D3 | | Threonine synthase, chloroplast | 1.31 | -4.62 | 4q24 |
| NM_017866 | | | TMEM70 | | Transmembrane protein 70 | 1.31 | -4.59 | 8q21 |
| NM_016048 | | | ISOC1 | | Isochorismatase domain containing 1 | 1.29 | -4.40 | 5q23 |
| NM_004048 | | | B2M | | Beta-2-microglobulin | 1.25 | -2.66 | 15q21 |
| NM_033546 | | | MRLC2 | | Myosin regulatory light chain MRLC2 | 1.23 | -1.85 | 18 p11 |
| NM_173515 | | | CNKSR3 | | CNKSR family member 3 | 1.22 | -3.48 | 6q25 |
| NM_003640 | | | IKBKAP | | Inhibitor of kappa light polypeptide gene enhancer in B-cells, kinase complex-associated protein | 1.22 | -3.41 | 9q31 |
| NM_000318 | | | PXMP3 | | Peroxisomal membrane protein 3, 35kDa | 1.22 | -6.62 | 8q21 |
| NM_005437 | | | NCOA4 | | Nuclear receptor coactivator 4 | 1.22 | -3.91 | 10q11 |
| NM_014267 | | | SMAP | | Small acidic protein | 1.21 | -5.66 | 11p15 |
| NM_138446 | | | C7orf30 | | Chromosome 7 open reading frame 30 | 1.21 | -2.55 | 7p15 |
| NM_016355 | | | DDX47 | | DEAD (Asp-Glu-Ala-Asp) box polypeptide 47 | 1.20 | -2.93 | 12p13 |
| NM_001449 | | | FHL1 | | Four and a half LIM domains 1 | 1.19 | -15.11 | Xq26 |
| NM_000274 | | | OAT | | Ornithine aminotransferase (gyrate atrophy) | 1.19 | -7.39 | 10q26 |
| NM_016284 | | | CNOT1 | | CCR4-NOT transcription complex, subunit 1 | 1.18 | -2.92 | 16q21 |
| NM_001018111 | | | PODXL | | Podocalyxin-like | 1.18 | -9.66 | 7q32 |
| NM_000712 | | | BLVRA | | Biliverdin reductase A | 1.18 | -2.50 | 7p13 |
| NM_019029 | | | CPVL | | Carboxypeptidase, vitellogenic-like | 1.17 | -15.50 | 7p15 |
| NM_001008897 | | | | TCP1 | T-complex 1 | 1.17 | -7.27 | 6q25 |
| NM_006708 | | | GLO1 | | Glyoxalase I | 1.17 | -2.64 | 6 p21 |
| NM_002881 | | | RALB | | v-ral simian leukemia viral oncogene homolog B (ras related; GTP binding protein) | 1.16 | -2.20 | 2q14 |
| NM_017750 | | | RetSat | | All-trans-13,14-dihydroretinol saturase | 1.16 | -2.44 | 2p11 |
| NM_016324 | | | ZNF274 | | Zinc finger protein 274 | 1.16 | -2.76 | 19q13 |
| NM_002802 | | | PSMC1 | | Proteasome 26S subunit, ATPase, 1 | 1.16 | -1.71 | 14q32 |
| NM_024077 | | | SECISBP2 | | SECIS binding protein 2 | 1.15 | -3.12 | 9q22 |
| NM_006430 | | | CCT4 | | Chaperonin containing TCP1, subunit 4 (delta) | 1.15 | -4.76 | 2p15 |
| NM_000454 | | | SOD1 | | Superoxide dismutase 1, soluble | 1.11 | -3.54 | 21q22 |
| NM_006367 | | | CAP1 | | CAP, adenylate cyclase-associated protein 1 | 1.10 | -5.62 | 1p34 |
| NM_002793 | | | PSMB1 | | Pproteasome subunit, beta type, 1 | 1.10 | -4.54 | 6q27 |
| NM_007167 | | | ZMYM6 | | Zinc finger, MYM-type 6 | 1.10 | -4.83 | 1p34 |
| NM_001025 | | | RPS23 | | Ribosomal protein S23 | 1.06 | -2.45 | 5q14 |
| NM_002136 | | | HNRPA1 | | Heterogeneous nuclear ribonucleoprotein A1 | 1.04 | -3.63 | 12q13 |
| NM_001914 | | | CYB5 | | Cytochrome b-5 | -1.10 | -2.84 | 18q22 |
| NM_022766 | | | CERK | | Ceramide kinase | -1.12 | -2.72 | 22q13 |
| NM_024026 | | | MRP63 | | Mitochondrial ribosomal protein 63 | -1.14 | -0.88 | 13q12 |
| NM_000378 | | | WT1 | | Wilms tumor 1 | -1.17 | -1.61 | 11p13 |
| XM_166164 | | | LOC219854 | | Hypothetical protein LOC219854 | -1.18 | -1.42 | 11q24 |
| NM_019843 | | | EIF4ENIF1 | | Eukaryotic translation initiation factor 4E nuclear import factor 1 | -1.19 | -2.14 | 22q12 |
| NM_001457 | | | FLNB | | Filamin B, beta (actin binding protein 278) | -1.23 | -2.04 | 3p14 |
| NM_002998 | | | SDC2 | | Syndecan 2 (heparan sulfate proteoglycan 1, cell surface-associated, fibroglycan) | -1.23 | -1.95 | 8q22 |
| NM_001390 | | | DTNA | | Dystrobrevin, alpha | -1.24 | -8.25 | 18q12 |
| NM_005194 | | | CEBPB | | CCAAT/enhancer binding protein (C/EBP), beta | -1.25 | -1.81 | 20q13 |
| NM_001014842 | | | TM9SF1 | | Transmembrane 9 superfamily member 1 | -1.25 | -1.50 | 14q11 |
| NM_031866 | | | FZD8 | | Frizzled homolog 8 (Drosophila) | -1.29 | -0.80 | 10p1 |
| NM_001015881 | | | TSC22D3 | | TSC22 domain family, member 3 | -1.29 | -4.60 | Xq22 |
| NM_004853 | | | STX8 | | Syntaxin 8 | -1.31 | -2.34 | 17p13 |
| NM_018434 | | | RNF130 | | Ring finger protein 130 | -1.41 | -8.53 | 5q35 |
| NM_025155 | | | WDR71 | | WD repeat domain 71 | -1.41 | -2.13 | 11q13 |
| NM_005407 | | | SALL2 | | Sal-like 2 (Drosophila) | -1.45 | -3.98 | 14q11 |
| NM_014767 | | | SPOCK2 | | Sparc/osteonectin, cwcv and kazal-like domains proteoglycan (testican) 2 | -1.49 | -3.23 | 10q22 |
| NM_032314 | | | MGC4767 | | Hypothetical protein MGC4767 | -1.50 | -1.45 | 12q24 |
| NM_005769 | | | CHST4 | | Carbohydrate (N-acetylglucosamine 6-O) sulfotransferase 4 | -1.53 | -0.31 | 16q22 |
| NM_022138 | | | SMOC2 | | SPARC related modular calcium binding 2 | -1.59 | -4.48 | 6q27 |
